# ADHD in adults with major depressive or bipolar disorder: does it affect clinical features, comorbidity, quality of life, and global functioning?

**DOI:** 10.1186/s12888-022-04273-8

**Published:** 2022-11-15

**Authors:** Mohammad Reza Sadeghian Nadooshan, Zahra Shahrivar, Javad Mahmoudi Gharaie, Leyla Salehi

**Affiliations:** Department of Psychiatry, Roozbeh Hospital, Tehran University of Medical Sciences, Tehran, Iran

**Keywords:** Adult ADHD, Depression, Bipolar, Comorbidity, Function, Quality of life

## Abstract

**Background:**

This study compared clinical characteristics, concurrent disorders, level of function, and quality of life in adults with bipolar (BD) or major depressive disorder (MDD) in those with/without adult attention defici1t hyperactivity disorder (AADHD).

**Methods:**

The participants were recruited among adult inpatients and outpatients with MDD or BD in their current partial remission in a psychiatric hospital. They were evaluated using the interview for adults with ADHD (DIVA-5), Conners’ Adult ADHD Rating Scales–Self-Report-Screening Version (CAARS-SR-SV), Structured Clinical Interview for DSM-V (SCID-5), Beck Depression Inventory-II and Young Mania Rating Scale, Global Assessment of Functioning (GAF) and World Health Organization Quality of Life Scale-Brief (WHOQoL-BREF).

**Results:**

In those with MDD (n = 105) and BD (n = 103), AADHD was detected as 13.3% and 16.5%, respectively. The inattentive presentation was the most prevalent type among patients with AADHD. Compared to the patients without AADHD, the results regarding the prevalence of comorbidities, suicidal attempts, severity of affective episodes, the early emergence of the affective disorders, and level of quality of life and global functioning were poorer in the group with AADHD (p < 0.05).

**Limitations:**

The participants were individuals with major depressive or bipolar type I disorder with a mostly manic episode, chosen among the referrals to a tertiary psychiatric hospital with high comorbidity and more severe psychopathology. This may limit the generalizability of the findings.

**Conclusion:**

ADHD was common in adults with MDD and BD, along with high psychiatric comorbidity and negative consequences. Clinicians are suggested to screen adults with mood disorders for the symptoms of ADHD for a more precise and comprehensive diagnosis and to provide a more appropriate therapeutic intervention.

## Introduction

Attention-deficit hyperactivity disorder (ADHD), a common disabling mental health condition in adults, is reported to be prevalent up to 5% in the general population[1, 2] .with a high rate of comorbid psychiatric disorders, such as anxiety, mood, learning, eating, substance use and personality disorders[3].

Research shows a great burden of ADHD on different and multiple domains of the affected individuals’ life including family relations, occupational environment, and academic achievements. This especially impacts their career and education and makes them prone to risky behaviors, including addiction. People with ADHD face many difficulties and challenges in their interpersonal and social relationships due to their core symptoms or other concurrent deficits and problems such as poor social skills. These factors together lessen their well-being and quality of living[4].

Most adults with ADHD (AADHD) are not recognized and remain untreated, however many of them are referred and receive treatment for other comorbid mental disorders, including major depressive disorder (MDD). The high comorbidity of mental disorders, including MDD with this neurodevelopmental disorder (80%) suggests a probable different picture of the clinical characteristics of AADHD in depressed adults[2]. A greater risk of MDD is reported in adolescents with ADHD, while the overlap between the two disorders in terms of their cognitive symptoms makes the differential diagnosis difficult[5]. The prevalence of co-occurring ADHD in individuals with MDD is 5–12%, a condition that leads to more severe symptoms and a negative prognosis[5–7].

ADHD is also commonly (10–20%) reported in adults with bipolar I disorder (BD) [7–9]. In adults suffering from BD and comorbid ADHD, an earlier age of onset, higher frequency of mood episodes, greater occurrences of suicidal attempts, and more probability of being diagnosed as anxiety disorders (AD) are observed[10].

AADHD is mentioned to be a risk factor for depression in adults experiencing stress, adjustment issues, and academic and social problems. Moreover, it can increase the duration of depressive episodes in patients with BD. Adults with co-occurrence of ADHD and MDD are at higher risk of diagnostic change to BD in comparison with those who have MDD alone[11].

Adults with ADHD and comorbid mood disorders may be at risk for misdiagnosis. Many clinicians are not expert enough to distinguish ADHD in adults suffering from other psychiatric problems including affective disorders. Besides, mood symptoms including depression cover the core manifestations of comorbid disorders such as attention deficit and hyperactivity, leading to being treated only for mood disorders, and not for ADHD[12].

It has been reported that only 11% of adults with ADHD receive treatment. One of the reasons for this undertreatment is its comorbidity with other psychiatric disorders including depressive and bipolar disorders[13].

These mood disorders, especially bipolar conditions, lead to underdiagnosis of AADHD in individuals and put them at risk of maltreatment, losing the chance of receiving the proper pharmacologic and non-pharmacologic interventions. People with concurrent AADHD and major mood disorders, without receiving appropriate treatment, are prone to face major health problems and negative life consequences[14].

Given the importance of AADHD and its comorbidity with mood disorders that are associated with a high likelihood of underdiagnosis and undertreatment, this study was aimed to evaluate the clinical characteristics and symptom severity of ADHD in a clinical group of adults with MDD or BD. Besides, considering the burden of ADHD in daily living[4], we decided to assess the global level of functioning, and quality of life in those with mood disorders and ADHD compared to the individuals without ADHD. We hoped that the findings would help to identify ADHD in those with mood disorders and complex presentations.

## Materials and methods

### Participants and Procedure

This cross-sectional study was conducted among outpatient and admitted referrals to Roozbeh psychiatric hospital, a tertiary university-affiliated center in Tehran, the capital of Iran from January 2018 to February 2019. It should be mentioned that most inpatients at adult wards are those who suffer from acute manic or mixed episodes of bipolar mood disorders, affective or non-affective psychotic disorders, and substance use-related mood or psychotic disorders. Among the outpatient referrals, there are a significant number of those previous inpatients with major psychiatric disorders who pursue the follow-up of their psychiatric intervention. Most individuals with bipolar or unipolar depressive disorders are not admitted and receive outpatient treatment.

The inclusion criteria were: age between18 and 65 years, being diagnosed as MDD or BD (type I), negative history of any cognitive disorders (e.g., dementia), or intellectual disability. In the case of delirium or acute intoxication/withdrawal of substance, the assessments were postponed to a later time when the participant was ready to cooperate with the study assessments. Two male patients with BD were excluded because of early voluntary discharge from the psychiatric ward.

A resident of psychiatry weekly checked new admissions to psychiatric wards for patients with a current episode of mood disorders. He prepared a primary checklist of those who had the inclusion criteria and followed their progress based on the responsible psychiatrist’s notes and nurses’ reports. All participants who were enrolled in the study were thoroughly evaluated in the current partial remission from the acute symptoms based on the psychiatric resident’s clinical judgment. He enrolled the patients when (a) at least 30% of the manic or depressive symptoms subsided; (b) the participants had the partial insight to the condition, and (c) they were ready to cooperate with the study assessments. The time of assessment was mostly near the discharge from the hospital. However, for less than 20% of the individuals, he set a schedule for an appropriate time after discharge which was less than one month. Moreover, he recruited some of the participants among those outpatients who attended the clinic for their follow-up sessions.

Among those with MDD, 51.5% were recruited from inpatients (30 females, 24 males), while the others (48.5%) were outpatients (22 females, 29 males). Those with BD were also selected from inpatients (82%, 38 females, 48 males) and outpatient clinics (18%, 11 females, and 8 males).

After an explanation of the aims and process of the study, the participants enrolled if they gave informed consent. A questionnaire was completed to check the demographic and clinical characteristics of each participant. For all the participants, the diagnosis of MDD and BD was done by a board-certified psychiatrist based on the DSM-V criteria as the routine clinical assessment of the ward or clinic. Then the Structured Clinical Interview for DSM-V Axis I Disorders (SCID-5) was used to confirm the diagnosis and check for other comorbidities. To screen for ADHD symptoms, the participants were asked to complete the Conner’s Adult ADHD Rating Scale-Self-Report: Screening Version. For those who had a score of 65 or higher, the semi-structured Diagnostic Interview for Adult ADHD (DIVA-5) was performed by a trained resident of psychiatry to check if the criteria of AADHD were fulfilled. To ensure the accuracy of the information, if any of the family members were available, they were asked to come to the hospital and accompany the participant during the DIVA-5 interview. The participants were asked to complete the World Health Organization Quality of Life Scale-Brief (WHOQOL BREF) to assess their quality of life. Moreover, the Beck Depression Inventory-II self-report and The Young Mania Rating Scale were rated by the participants with depressive disorder (MDD or bipolar depression) and BD, respectively to assess the severity of mood symptoms. The Global Assessment of Functioning (GAF) was also completed to rate the level of general functioning by the psychiatry resident. Finally, those with and without AADHD were compared based on their mood symptoms, quality of life, global functioning, and other clinical and demographic characteristics.

## Instruments

### Conner’s adult ADHD rating scale- Self-report: Screening Version (CAARS-SR-SV)

The CAARS-S: S provides a useful dimensional evaluation system for both research and clinical use. It is a 26-item questionnaire, rated on a Likert-type scale (0 = not at all, 3 = severe) according to the patients’ current functional status[15]. Davari-Ashtiani et al. (2014) validated the Persian version of the CAARS-SR: SV and found the Cronbach’s alpha coefficient of all the subscales to be higher than 0.8.

### Structured clinical interview for DSM-V Axis I Disorders (SCID-5)

The SCID-5 is a widely used semi-structured diagnostic interview to assess the presence of mental disorders based on the DSM-V criteria for axis I disorders. The interview takes approximately 1 h to complete[16]. There is no psychometrics on SCID-5 in Iran yet, however, Sharifi et al. (2009) conducted the Persian version of SCID-I in a multi-center study and supported its validity for clinical and especially research purposes.

### Beck Depression Inventory-II self-report

The BDI-II SR was administered as a self-report questionnaire, consisting of 21 items ranging from 0 to 3 points[17]. The total score ranges from 0 to 63 with a higher score as more severe symptoms. The validity and reliability of the BDI-II in an Iranian sample were reported being acceptable[18].

*Young Mania Rating Scale (YMRS)*.

This is an 11-item scale, with a total score from 0 to 60 [19]. Using the patient report and the clinician observation the items are rated regarding the manic symptoms. The validity and reliability of the YMRS were confirmed in Iran [20].

### Global Assessment of Functioning (GAF)

The GAF is a scale introduced to rate the level of functioning. It is a 100-point scale, with 10-point intervals. The most severely poor functioning is described with 1–10 and the greatest with the score interval of 91–100[21, 22].

### Diagnostic interview for adult ADHD (DIVA-5)

It is a semi-structured diagnostic interview to assess ADHD in adults based on the diagnostic criteria of DSM-V. The DIVA has been reported to be a reliable diagnostic tool for clinical and research purposes[23]. The psychometric properties of the Persian-version of DIVA have been supported in Iran [24].

### World Health Organization Quality of life scale-brief (WHOQOL BREF)

This is a 26-item questionnaire[25] extracted from the 100-item World Health Organization Quality of Life Scale. It requires assessing 4 fields of quality of life. Each item is scored on a 5-point Likert Scale. Nejat et al. (2006) conducted the Persian version of WHOQOL BREF and supported its reliability and validity[26].

### Statistical analysis

Descriptive statistics were expressed in means and standard deviations or percentages as appropriate. Differences in characteristics between the participants were assessed by independent t-tests. Prevalence of AADHD and its subtypes, and other Axis I disorders were estimated for the total sample and each gender separately. Chi-square was used to investigate the relationship between qualitative data. The Correlation Coefficient was applied to consider the association among the study variables. P-values were considered significant at the level of p < 0.05. Data were analyzed using the IBM SPSS Statistics 24.0.

## Results

A total of 210 participants with mood disorders were enrolled in the study including 105 patients with MDD (50.5% males, 26.7% single, mean age = 39.89 years), and 105 patients with type I BD (53.3% males, 46.7% single, mean age = 34.37 years). Among those with BD diagnosis, 88 individuals were diagnosed with a manic episode (36 females and 38 males were inpatients, 7 females and 7 males were outpatients), and 17 patients suffered from a depressive episode (2 females and 10 males were inpatients and 4 females and 1 male outpatient).

No significant differences were found between the groups regarding the demographic variables. Table 1 represents the findings on the comparison of the demographic characteristics between the two groups.

**Table 1:** Demographic characteristics of the participants with and without AADHD in the two groups with MDD or BD

Participants	*MDD		P	**BD		P
	+ AADHD*** (N = 14) (%)	- AADHD (N = 91) (%)		+ AADHD (N = 17) (%)	- AADHD (N = 86) (%)	
Gender						
Male	7(50%)	46(50.4%)	0.598	10(58.8%)	44(51.2%)	0.379
Female	7(50%)	45(49.6%)		7(41.2%)	42(48.8%)	
Education						
Unlettered	0(0%)	7(7.7%)	0.571	0(0%)	3(3.5%)	0.675
Under diploma	6(42.8%)	45(49.4%)		9(53%)	41(47.7%)	
Diploma	4(28.6%)	23(25.3%)		4(23.5%)	28(32.5%)	
Higher than diploma	4(28.6%)	16(17.6%)		4(23.5%)	14(16.3%)	
Job						
Unemployed	10(71.4%)	31(34.1%)	0.689	12(70.5%)	42(48.8%)	0.559
Self-employed	2(14.3%)	57(62.6%)		2(11.8%)	37(43.1%)	
Student	1(7.15%)	0(0%)		1(5.9%)	4(4.6%)	
Employee	1(7.15%)	3(3.3%)		2(11.8%)	3(3.5%)	
Marital Status						
Single	8(57.2%)	20(22%)	0.074	11(64.8%)	38(44.2%)	0.126
Married	3(21.4%)	51(56%)		3(17.6%)	37(43.1%)	
Divorced	3(21.4%)	18(19.8%)		3(17.6%)	7(8.1%)	
Separated	0(0%)	2(2.2%)		0(0%)	4(4.6%)	

*Major Depressive Disorder; **Bipolar I Disorder; ***Adult attention-deficit hyperactivity disorder

The mean severity of the manic symptoms in the group with BD episodes rated according to the Young Mania questionnaire (YMRS), was 17.29 ± 6.45.

The Beck questionnaire (BDI-II) was used to examine the severity of depressive symptoms in both groups of MDD and BD. These values were in a sequence of 16.26 ± 6.11 and 15.43 ± 5.56 in the two groups.

Among those with MDD and BD, 13.3% and 16.5% were diagnosed as AADHD respectively.

Among the group with BD, AADHD was only found in the individuals who had remitted from a manic episode. It means that none of those BD patients with depressive episodes were diagnosed as suffering from AADHD.

Based on the DSM 5 (2013) classification, individuals with ADHD can be categorized as having a ‘‘Hyperactivity and Impulsivity’’ or ‘‘Inattention’’ presentation. The first subtype is considered when the individuals have problems with sitting calmly, talkativeness, restlessness, and impulsive decision and behavior. The latter subtypes are characterized by difficulty in planning and a deficit in paying attention to details, and remembering what to do.

The inattentive and combined presentation was found in 57.2% and 42.8% of the participants with MDD + AADHD and 23.5% and 76.5% of the participants with BD + AADHD, respectively. There was a negative association between age and prevalence of AADHD in each group (MDD + AADHD; p = 0.008, BD + AADHD; p = 0.015).

In each group of MMD or BD, the participants with and without AADHD were compared with each other based on their clinical features (Table 2).

**Table 2:** Clinical features of the participants with and without AADHD in the two groups with MDD and BD separately

Participants	*MDD		P	**BD		P
	+ AADHD*** (N = 14) (%)	- AADHD (N = 91) (%)		+ AADHD (N = 17) (%)	- AADHD (N = 86) (%)	
Average age of onset of the disorder	22.64 ± 5.18	33.46 ± 10.32	**0.001**	19.18 ± 4.26	22.76 ± 6.66	**0.008**
Average number of suicide attempts	1.86 ± 1.79	0.97 ± 1.33	**0.094**	2.12 ± 1.76	0.84 ± 1.24	**0.001**
Average number of hospitalizations	2.29 ± 2.58	1.73 ± 1.49	0.245	3.29 ± 2.36	3.70 ± 3.47	0.648
Average number of depressive episodes	3.36 ± 2.02	2.54 ± 1.34	0.431	2.82 ± 1.33	3.27 ± 3.22	0.579
Average severity of depressive symptoms (BDI-II)	25.71 ± 6.34	14.85 ± 6.06	**0.001**	-----	-----	-----
Average severity of manic symptoms (YMRS)	-----	-----	-----	28.82 ± 4.87	14.46 ± 6.85	**0.001**

*Major Depressive Disorder; **Bipolar I Disorder; ***Adult attention-deficit hyperactivity disorder

Rate of comorbidity in all participants with MDD was 0 in 32.4%, 1 in 32.4%, 2 in 30.5%, 3 in 2.9%, 4 in 1%, and 5 in 1%. For the group with BD, this rate was 0 in 41%, 1 in 27.6%, 2 in 27.6%, and 3 in 3.8%. In the group with MDD, the mean number of the comorbid disorders was 2.28 and 0.92 in those with and without AADHD (p = 0.001). In the group with BD, the average range of concurrent disorders was 0.86 and 1.47 in the participants without and with AADHD, respectively (p = 0.012). Among the BD participants with AADHD, 88.2% had at least one comorbid disorder and 47.1% had more than 2 disorders. These rates in the participants with MDD + AADHD were 92.9% and 78.6%. Table 3 demonstrates the frequency of comorbid disorders in the participants with and without AADHD in each group with MDD or BD.

**Table 3:** Comorbidity with other psychiatric disorders in the participants with and without AADHD in the two groups with MDD and BD, separately

Participants	*MDD		P	**BD		P
	+ AADHD*** (N = 14) (%)	- AADHD (N = 91) (%)		+ AADHD (N = 17) (%)	- AADHD (N = 86) (%)	
Obsessive Compulsive Disorder	4(28.6%)	14(15.4%)	0.223	4(23.5%)	19(22.1%)	0.897
Generalized Anxiety Disorder	5(35.7%)	19(20.9%)	0.218	5(29.4%)	10(11.6%)	**0.058**
Dysthymia	4(28.6%)	5(5.5%)	**0.004**	0	0	0
Alcohol Use Disorder	0(0%)	3(3.3%)	0.491	4(23.5%)	2(2.3%)	**0.001**
Opiates Use Disorder	2(14.3%)	15(16.5%)	0.835	0(0%)	15(17.4%)	0.062
Cannabis Use Disorder	3(21.4%)	10(11%)	0.270	4(23.5%)	12(13.9%)	0.319
Amphetamine Use Disorder	1(7.1%)	2(2.2%)	0.301	0(0%)	4(4.6%)	0.364
Drug Use Disorder	4(28.6%)	8(7.6%)	**0.003**	5(29.4%)	8(9.3%)	**0.023**
Social Anxiety Disorder	2(14.3%)	1(1.1%)	**0.001**	0(0%)	1(1.2%)	0.655
Post-Traumatic Stress Disorder	2(14.3%)	3(3.3%)	0.720	0(0%)	1(1.2%)	0.655
Agoraphobia	0(0%)	3(3.3%)	0.491	2(11.8%)	0(0%)	**0.001**
Panic Disorder	0(0%)	2(2.2%)	0.575	2(11.8%)	1(1.2%)	**0.018**
Eating Disorder	1(7.1%)	1(1.1%)	0.124	1(5.9%)	2(2.3%)	0.426

*Major Depressive Disorder; **Bipolar I Disorder; ***Adult attention-deficit hyperactivity disorder

Rate of substance use disorders (SUD) in all participants with BD included 0 in 52.4%, 1 in 14.3%, 2 in 17.1%, 3 in 6.7%, 4 in 8.6% and 5 in 1%. This number for the group with MDD was 0 in 57.1%, 1 in 19%, 2 in 12.4%, 3 in 6.7%, 4 in 3.8% and 5 in 1%. The mean number of SUD in BD individuals with and without AADHD was 1.7 and 0.98, respectively (p = 0.063). For the individuals with MDD, these results were in a sequence of 1.2 and 0.88. Also, 64.1% of the BD group had cigarette smoking (88.2% in those with A-ADHD, p = 0.023). This rate was 49.5% in patients with MDD (71.4% in those with A-ADHD, p = 0.078).

Regarding the quality of life, the results on WHOQOL BREF mean scores were compared between the individuals with and without AADHD in each group of MDD or BD (Table 4).

**Table 4:** Quality of Life in the participants with and without AADHD in the two groups with MDD and BD, separately

Clinical features	*MDD		P	**BD		P
	+ AADHD*** (N = 14) (%)	- AADHD (N = 91) (%)		+ AADHD (N = 17) (%)	- AADHD (N = 86) (%)	
Physical	20.71 ± 2.23	25.08 ± 3.15	**0.001**	20.88 ± 3.55	25.13 ± 3.19	**0.001**
Psychic	15.28 ± 1.48	21.31 ± 3.02	**0.001**	15.17 ± 2.62	20.67 ± 3.06	**0.001**
Social	6.28 ± 0.72	8.96 ± 1.58	**0.001**	6.17 ± 0.95	8.82 ± 1.69	**0.001**
Environmental	22.14 ± 2.41	26.34 ± 2.38	**0.001**	21.05 ± 2.22	25.46 ± 3.30	**0.001**

*Major Depressive Disorder; **Bipolar I Disorder; ***Adult attention-deficit hyperactivity disorder

Table 5 shows the level of the participants’ global functioning based on the GAF scores in the two groups, comparing those with and without AADHD.

**Table 5:** Global assessment of functioning in the participants with and without AADHD in the two groups with MDD and BD, separately

Global function		-AADHD***		+AADHD		P
		number	%	number	%	
MDD*	21–30	0	0	1	7.1	0.001
	31–40	6	6.6	5	35.7	
	41–50	17	18.7	3	21.4	
	51–60	37	40.7	4	28.6	
	61–70	24	26.4	1	7.1	
	71–80	7	7.7	0	0	
BD**	21–30	1	1.2	3	17.6	0.001
	31–40	15	17.4	7	41.2	
	41–50	27	31.4	6	35.3	
	51–60	23	26.7	1	5.9	
	61–70	18	20.9	0	0	
	71–80	2	2.3	0	0	

*Major Depressive Disorder; **Bipolar I Disorder; ***Adult attention-deficit hyperactivity disorder

## Discussion

This study was conducted to evaluate the prevalence of adult ADHD (AADHD) in individuals with type I bipolar disorder (BD) or major depressive disorder (MDD) and to compare clinical features, global functioning, quality of life, and comorbidities in those with and without AADHD.

The prevalence rate of AADHD was 16.5% among the participants with BD in the current study, a rate up to 4 times higher than in a group of Iranian general population as a university student [27]. The reported rate of AADHD in individuals with BD by other studies was from 16.3–25%[8, 28–32], which most of them are higher than the prevalence in the present study. It is noteworthy that all of the above-mentioned studies used self-report questionnaires to check for AADHD, compared to the structured interview (DIVA-5) we used. Moreover, the study by Pinna et al. (2019) was a retrospective cohort that gathered the data based on a specialized clinician judgment[31].

Most participants with BD in the recent study were those with a chronic condition and a relapse of an acute episode of mania. The assessment of the severity of mania using the YMRS was done when they were still admitted (82%). The remaining individuals were evaluated within one month after discharge. The reported cut-off for YMRS in Iran is 17.5 [20]. The high mean scores of the individuals with BD in our study can be explained by the fact that most patients who are usually admitted to Roozbeh hospital suffer from severe symptoms of the disorder. Therefore, the high YMRS score at the time of assessment when they were in partial remission seems justifiable. When most participants’ mean scores are high, the scores after partial therapeutic response would be still higher than the cut-point. This was especially higher in those with AADHD compared to those without it.

In comparison with BD, the prevalence of AADHD in the patients with MDD in the present study was lower (13.3%), a rate close to the prevalence rate of 12.5% by Dunlop et al. (2018)

and 12% by Alpert et al. (1996) [5, 33].

The high prevalence of ADHD symptoms in adults with MDD or BD in the recent study is consistence with previous studies and underscores the importance of paying attention to this diagnosis when evaluating mood symptoms in the referrals.

The Association between demographic factors and the prevalence of AADHD was assessed in the two groups. We did not find any significant gender relations between the two groups. This is similar to many studies supporting an equal rate of ADHD in males and females with depressive or bipolar disorders[34]. However, consistent with Simon et al. (2013) and Faraone et al. (2006) studies, we found a significant negative relationship between age and the prevalence of AADHD in both groups of MDD and BD; the prevalence decreased with increasing age[35, 36]. Therefore, clinicians should pay attention to the comorbidity of ADHD diagnosis in younger adults with major mood disorders including MDD and BD.

In terms of marital status, although Pinna et al. (2019) showed a higher rate of divorce among those with AADHD compared to non-AADHD individuals in the general population, the present study did not find any significant relationship between AADHD prevalence and divorce in any of the two groups[31]. Some studies are showing that AADHD can affect family relationships leading to divorce or unmarried status[37]. However, the high percentage of single people and the low mean age of the participants in the current study could have affected these findings. We found no significant relationship between the education level and AADHD prevalence in any of the MDD or BD groups, which was consistent with the study by Amiri et al. (2010) on AADHD in the general population. However, this was not consistent with Murphy’s study[38].

We found a significant relationship between the age of onset of mood disorder and prevalence of AADHD in both groups of individuals with MDD or BD; the mean age at onset of the mood disorder was lower for those with AADHD. This finding was similar to Mclntyre et al. (2013), Nierenberg et al. (2005), Taman et al. (2008), and karaahmet et al. (2013) studies where the age-onset was about 5 years earlier compared to non-AADHD[7, 29, 32]. It has been suggested that ADHD makes people more vulnerable to other major psychiatric disorders, as secondary disorder or disorders with common inherited vulnerability [39, 40]. This important finding should be considered by mental health workers to screen for children and adolescents with attention-deficit and hyperactivity-impulsivity problems among their referrals. When they find those with the disorder, they can provide them with early interventions to prevent the negative consequences of the untreated problems, including secondary anxiety and mood conditions, and help them learn strategies for coping better with the syndrome.

The number of suicide attempt in those with BD + AADHD was significantly higher than in those with BD-AADHD in the current study, which is consistent with the results by Harmanci et al. (2016), Fili et al. (2019) and Pinna et al. (2019)[31, 41, 42]. Impulsivity as a trait can be assumed as an effective factor in attempting suicide for patients with ADHD compared to others without AADHD. However, this higher frequency of suicidal attempts was not replicated for the group with MDD + AADHD in our study. Contrary to our results, Dunlop et al. (2018) and Kawaki et al. (2012) showed that ADHD was an independent risk factor for suicidal ideation and behavior in individuals with MDD[5, 43]. By the way, suicide is a major concern that should be considered seriously in those with major mood disorders and/or AADHD. The findings suggest that when these disorders exist concurrently, the risk of self-harm is higher than each disorder alone.

We did not find any significant differences with regard to the number of hospitalizations or episodes of affective disorders in those with vs. without AADHD in any of the two groups. These results were inconsistent with most existing studies including Berkol et al. (2014) and Perugi et al. (2013) [28, 30]. It may be due to the severity of mood disorders in the participants in the recent study who were recruited among the referrals to a tertiary psychiatric hospital and needs further studies to find the reason. However, the higher comorbidity rates with other psychiatric disorders, the lower functional level, and the poorer quality of life in those with MDD or BD and AADHD compared to non-AADHD individuals were expected and replicated in the recent study.

In the present study, the severity of the affective disorder in both groups of depressed and bipolar disorders was significantly higher in the individuals with AADHD vs. without AADHD, based on the BDI-II and Young MRS mean scores, respectively. A study by Molavi et al. (2011) in adolescents with BD admitted to the Roozbeh psychiatric hospital, found greater severity of manic episodes and a lower overall recovery rate at 6-month follow-up in those with vs. without ADHD[44]. Pehlivanidis et al. (2014) examined ADHD comorbid with depressive and anxiety disorders, concluding that the patients with more severe symptoms should be screened for AADHD[45]. Hence, our findings support the importance of considering AADHD when the mood disorders are very severe and make the individuals much more dysfunctional and deteriorated.

The higher rate of concurrent psychiatric disorders among those who suffered from AADHD in both groups of patients with BD or MDD was consistent with other studies supporting the greater prevalence of concurrent psychopathology in patients diagnosed with AADHD[46]. Obsessive-compulsive disorder (OCD), substance use disorder (SUD), and generalized anxiety disorder (GAD) were more prevalent in patients with BD + AADHD, while in those with MDD + AADHD, GAD, OCD, and SUD were the most common conditions. These findings confirm the need to screen individuals with mood disorders for probable comorbid AADHD and concurrent psychiatric conditions to understand better the diagnostic problems and resolve the therapeutic challenges.

Opiates and cannabis were the most common substances used by the patients in the MDD and BD groups, respectively. Opiates were the most common substances used by those with MDD followed by cannabis and alcohol. For those with BD, cannabis was the most common followed by opiates and alcohol. Regarding the use of sedative-hypnotic drugs among both groups of MDD and BD, those with AADHD used more than the non-AADHD group. It was suggested that individuals with AADHD have a higher level of anxiety, representing sedatives use as an attempt to self-medicate the symptoms[47]. However, regarding the prevalence of SUD, we did not find any significant difference between the patients with and without AADHD among the MDD or BD groups. This finding is in accordance with Sadeghi Movahed et al. (2012) and Mihan et al. (2018), and inconsistent with some other studies [3, 6, 9, 38, 48, 49]. It seems that other potential factors may contribute to the prevalence of SUD in adults with comorbid ADHD and mood disorders, this issue needs more research to be clarified.

According to previous studies reporting a lower level of functioning in patients with AADHD than those without it [47, 49], lower GAF scores were found for the individuals diagnosed as AADHD in both groups of MDD and BD in the recent study. This finding was similar for each of the 4 domains of quality of life. Matthies et al. also showed the impact of depression on quality of life in participants with AADHD[50]. It can be concluded that concurrence of AADHD with each of MDD or BD in adults compared with mood disorders without this neurodevelopmental condition, increases the burden of the disorders on the quality of their life and decreases the level of their functioning in different domains of living. The notion should make the families, clinicians, health politicians, and stakeholders vigilant to the many important negative consequences of AADHD for those suffering from it, as well as for their relatives and communities they are living with.

## Strengths and Limitations

The present study used structured interviews as well as self-report instruments for a careful examination of the clinical diagnosis of adult ADHD and other psychiatric disorders in a clinical group of both outpatient and inpatient participants with MDD or BD. However, the findings should be considered in light of some limitations.

The participants were recruited among clinical referrals to a psychiatric hospital where most were suffering from severe psychopathology, hence the results are not generalizable to community samples. Moreover, the assessments were multiple and time-consuming. Therefore, the researcher tried to match the best time for the clinical evaluation according to the patient’s schedules. However, it was not possible for some individuals especially the outpatients to attend the hospital for the next appointments, leading to attrition of some of them. Besides, we enrolled only the participants with type I bipolar disorder, hence the results cannot be generalized to other types of BD. The individuals who remitted partially from mania were almost five folds of whom remitted from a depressive episode of BD. This may affect the results when comparing the groups with and without AADHD.

## Conclusion

This study showed a high prevalence of AADHD in the participants with major depressive and bipolar disorders, supporting the fact that ADHD diagnosis in adults with major mood disorders has an important role in the disability of the patients including the decline in quality of life and global functioning level. Besides, the existence of ADHD in those adults with MDD and BD suggests a higher rate of other psychiatric disorders, especially OCD, GAD, and SUD. This fact underscores the importance of paying more attention to symptoms of ADHD in this group of individuals, as a necessary practice for a more comprehensive treatment, and to prevent related complications and improve the prognosis. Future research is needed to assess whether the treatment of ADHD can reduce its cost to life and the economy for individuals suffering from depressive or bipolar mood disorders, and improve their real-world outcomes for them.

## Data Availability

The datasets used and/or analysis during the current study are available from the corresponding author upon reasonable request.

## Declarations

All methods of this study were carried out in accordance with the Institutional Review Board (IRB) of Roozbeh Psychiatric Hospital (a tertiary university-affiliated center in Tehran). Ethical clearance was approved by the Ethics Committee of Tehran University of Medical Sciences, with reference number: IR.TUMS.MEDICINE.REC.1396.3487.

The study did not intervene in the medication or non-pharmacologic treatments being provided to the participants. After an explanation of the aims and process of the study, the participants enrolled if they gave informed consent. Written informed consent was obtained from all individual or guardian participants. The participants were allowed to quit the study whenever they wanted and their information was confidential.

## Abbreviations

BD: bipolar disorder.
MDD: major depressive disorder.
AHDH: attention defici1t hyperactivity disorder.
AADHD: adult attention defici1t hyperactivity disorder.
CAARS-SR-SV: Conners’ Adult ADHD Rating Scales–Self-Report-Screening Version.
SCID-5: Structured Clinical Interview for DSM-V.
GAF: Global Assessment of Functioning.
WHOQoL-BREF: World Health Organization Quality of Life Scale-Brief.
